# Supplementary figures and images for: The optimization of electrochemical hydride generation technology for treating antimony-containing wastewater
Source: PLoS One. 2025 Sep 4;20(9):e0331138. doi: 10.1371/journal.pone.0331138 (PMC12410798; doi:10.1371/journal.pone.0331138)

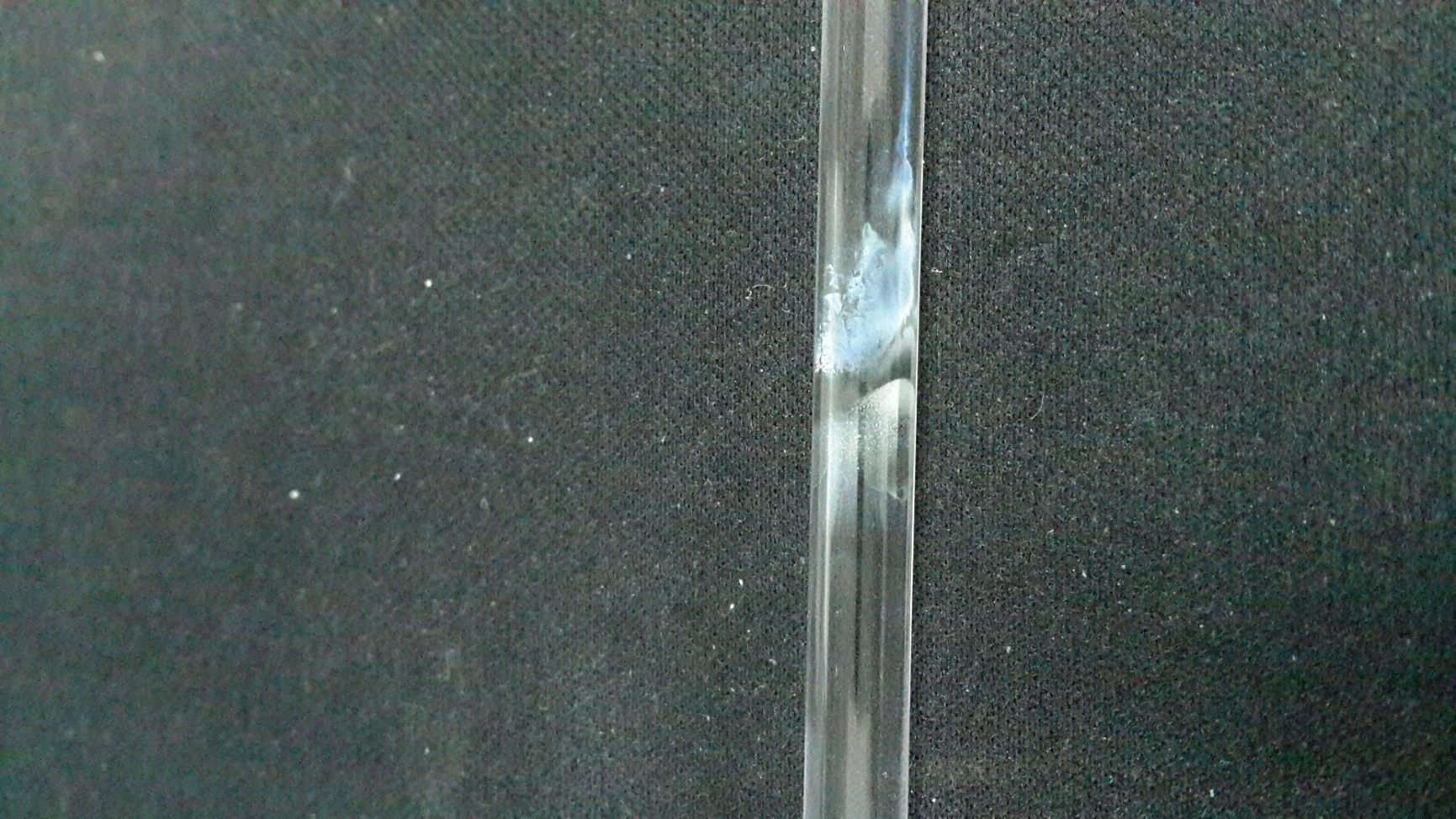

Supplement: S1 Fig — (TIF) [file pone.0331138.s001.tif]

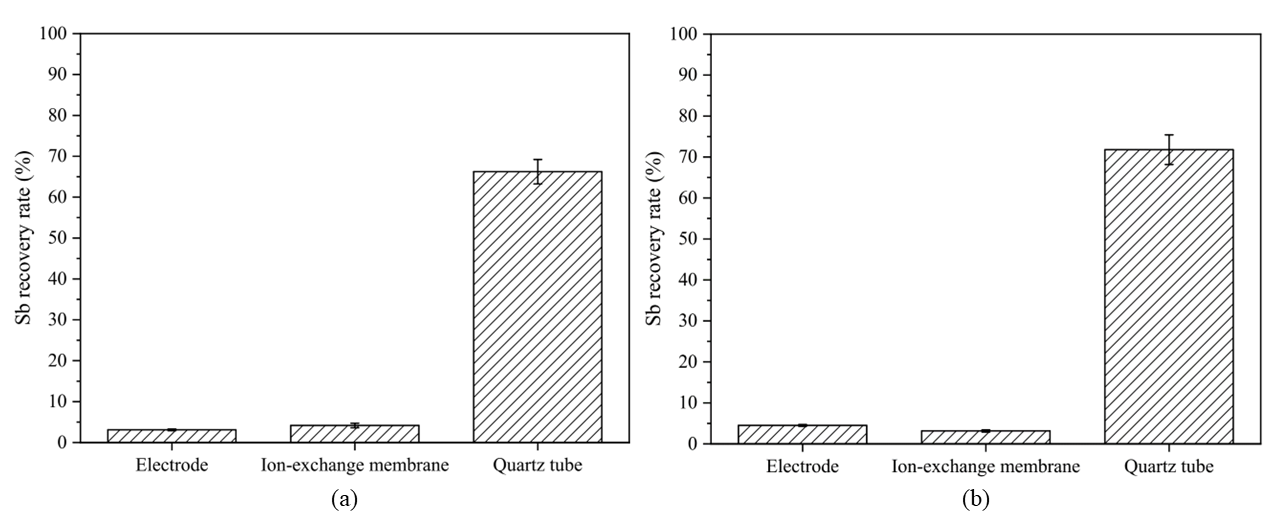

Supplement: S2 Fig — (TIF) [file pone.0331138.s002.tif]

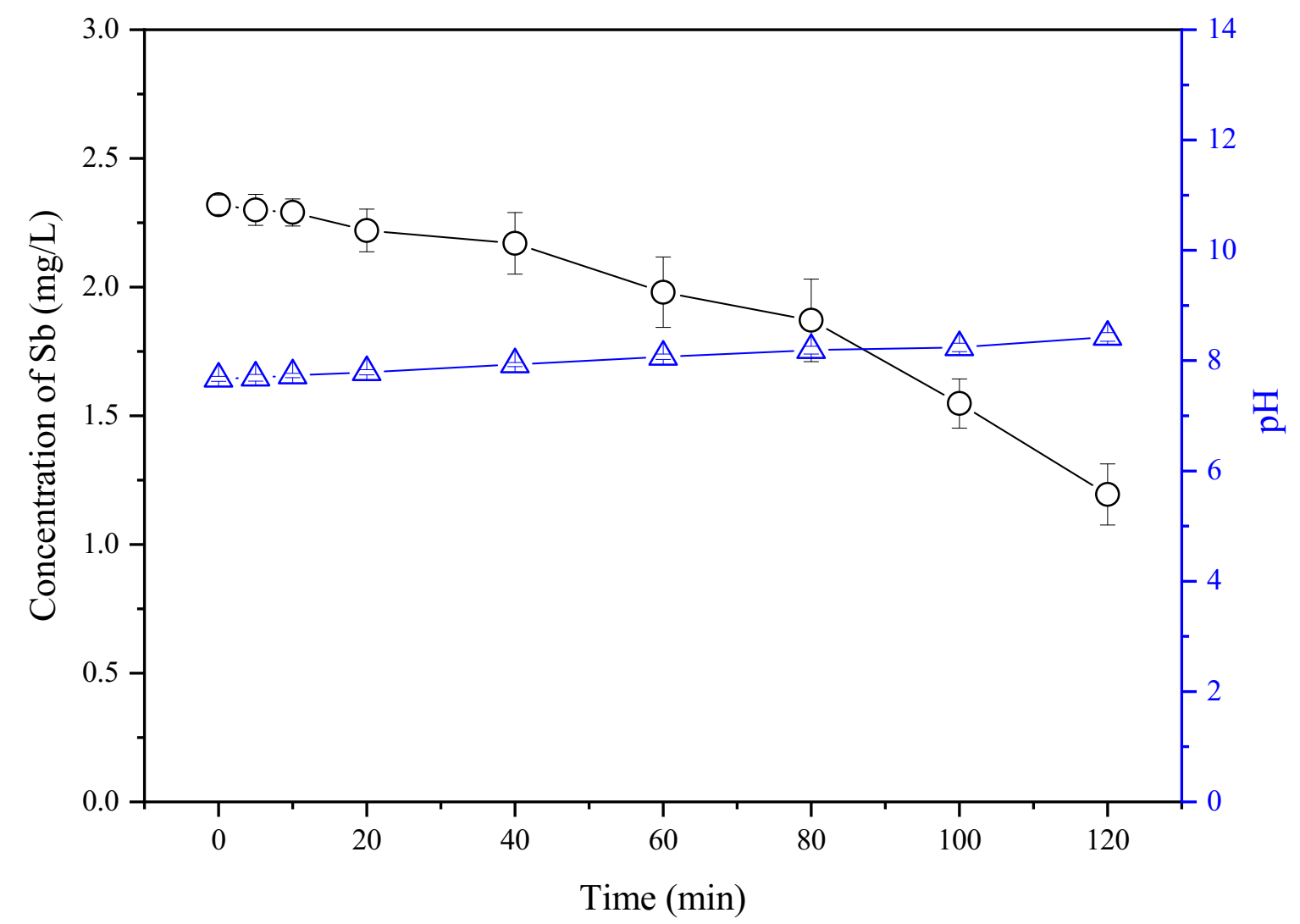

Supplement: S3 Fig — (TIF) [file pone.0331138.s003.tif]
