# Supplementary material for: The optimization of electrochemical hydride generation technology for treating antimony-containing wastewater
Source: PLoS One. 2025 Sep 4;20(9):e0331138. doi: 10.1371/journal.pone.0331138 (PMC12410798; doi:10.1371/journal.pone.0331138)
Supplement: S1 Table — (DOCX) [file pone.0331138.s004.docx]

**S1 Table. ANOVA for quadratic model.**

| Source | Model | A | B | AB | A² | B² |
| --- | --- | --- | --- | --- | --- | --- |
| Sum of Squares | 410.77 | 89.22 | 137.94 | 79.88 | 41.04 | 62.68 |
| df | 5 | 1 | 1 | 1 | 1 | 1 |
| F-value | 53.53 | 58.13 | 89.88 | 52.05 | 26.74 | 40.84 |
| p-value | < 0.0001 | < 0.0001 | < 0.0001 | < 0.0001 | 0.0006 | 0.0001 |

A-Current intensity；B-Electrode area
