# Supplementary material for: The optimization of electrochemical hydride generation technology for treating antimony-containing wastewater
Source: PLoS One. 2025 Sep 4;20(9):e0331138. doi: 10.1371/journal.pone.0331138 (PMC12410798; doi:10.1371/journal.pone.0331138)
Supplement: S2 Table — (DOCX) [file pone.0331138.s005.docx]

**S2 Table. Fit statistics.**

| Std. Dev. | Mean | C.V. % | R² | Adjusted R² | Predicted R² | Adeq Precision |
| --- | --- | --- | --- | --- | --- | --- |
| 1.24 | 70.31 | 1.76 | 0.9675 | 0.9494 | 0.8817 | 21.1495 |
