# Supplementary material for: The optimization of electrochemical hydride generation technology for treating antimony-containing wastewater
Source: PLoS One. 2025 Sep 4;20(9):e0331138. doi: 10.1371/journal.pone.0331138 (PMC12410798; doi:10.1371/journal.pone.0331138)
Supplement: S3 Table — (DOCX) [file pone.0331138.s006.docx]

**S3 Table. The impact of optimized conditions on Sb removal.**

|  | Optimum condition | Effectiveness | | |
| --- | --- | --- | --- | --- |
|  |  | Accelerate the removal of Sb | Improve the final removal rate of Sb | Reduce condition control |
| Membrane selection | Nafion | — | ⚫ | ⚫ |
| Stirring Method | Ultrasonic | ⚫ | — | — |
| Temperature | 30℃ | — | ⚫ | — |
| Current Intensity and Electrode Area | 0.5A，20cm^2^ | — | ⚫ | — |

⚫-Effect；—-No effect
