# Supplementary material for: The optimization of electrochemical hydride generation technology for treating antimony-containing wastewater
Source: PLoS One. 2025 Sep 4;20(9):e0331138. doi: 10.1371/journal.pone.0331138 (PMC12410798; doi:10.1371/journal.pone.0331138)
Supplement: S4 Table — (DOCX) [file pone.0331138.s007.docx]

**S4 Table. Compositional comparison of actual wastewater versus synthetic wastewater.**

|  | Actual wastewater | Synthetic wastewater |
| --- | --- | --- |
| pH | 7.76 | 6.69 |
| Fe(T) (mg/L) | 1.45 | 0 |
| Sb(T) (mg/L) | 2.32 | 5 |
| Sb(Ⅲ) (mg/L) | 0.73 | 5 |
| Sb(Ⅴ) (mg/L) | 1.59 | 0 |
| Sb removal (%) | 48.7 ± 5.4 | 71.8 ± 2.2 |
